# Transcriptional changes and developmental abnormalities in a zebrafish model of myotonic dystrophy type 1

**DOI:** 10.1242/dmm.012427

**Published:** 2013-10-02

**Authors:** Peter K. Todd, Feras Y. Ackall, Junguk Hur, Kush Sharma, Henry L. Paulson, James J. Dowling

**Affiliations:** 1 Department of Neurology, University of Michigan, Ann Arbor, MI 48109, USA.; 2 Department of Pediatrics, University of Michigan, Ann Arbor, MI 48109, USA.

**Keywords:** Muscleblind, CUG repeat, Nucleotide repeat, Neurodegeneration

## Abstract

Myotonic dystrophy type I (DM1) is a multi-system, autosomal dominant disorder caused by expansion of a CTG repeat sequence in the 3′UTR of the *DMPK* gene. The size of the repeat sequence correlates with age at onset and disease severity, with large repeats leading to congenital forms of DM1 associated with hypotonia and intellectual disability. In models of adult DM1, expanded CUG repeats lead to an RNA toxic gain of function, mediated at least in part by sequestering specific RNA splicing proteins, most notably muscleblind-related (MBNL) proteins. However, the impact of CUG RNA repeat expression on early developmental processes is not well understood. To better understand early developmental processes in DM1, we utilized the zebrafish, *Danio rerio*, as a model system. Direct injection of (CUG)_91_ repeat-containing mRNA into single-cell embryos induces toxicity in the nervous system and muscle during early development. These effects manifest as abnormal morphology, behavioral abnormalities and broad transcriptional changes, as shown by cDNA microarray analysis. Co-injection of zebrafish *mbnl2* RNA suppresses (CUG)_91_ RNA toxicity and reverses the associated behavioral and transcriptional abnormalities. Taken together, these findings suggest that early expression of exogenously transcribed CUG repeat RNA can disrupt normal muscle and nervous system development and provides a new model for DM1 research that is amenable to small-molecule therapeutic development.

## INTRODUCTION

Myotonic dystrophy type I (DM1) is the third most common muscular dystrophy, affecting an estimated one in 10,000 people worldwide (Bird, 2007). It is characterized clinically by effects in multiple organ systems, including muscle, heart and the central nervous system. DM1 is an autosomal dominant disorder that results from expansion of a non-coding CTG repeat in the 3′ untranslated region of the *DMPK* gene on chromosome 19 (Aslanidis et al., 1992; Brook et al., 1992; Harley et al., 1992; Mahadevan et al., 1992). The CUG repeat expansion as mRNA is able to bind to and sequester specific proteins, most notably the muscleblind-like protein family of splicing factors (MBNL1, MBNL2 and MBNL3) (Miller et al., 2000; Mankodi et al., 2001). This sequestration is thought to trigger altered splicing and expression of MBNL target mRNAs, which in turn result in the clinical symptoms observed in patients (Mankodi et al., 2000; Kanadia et al., 2003a; Jiang et al., 2004; Kanadia et al., 2006; Lin et al., 2006; Wheeler et al., 2007; Osborne et al., 2009; Du et al., 2010; Wang et al., 2012).

One striking feature of DM1 is the high degree of genetic anticipation that occurs over subsequent generations (Harper, 1975). Mothers who are only mildly affected clinically can give birth to children with very large CTG repeat expansions (typically greater than 2000 CTGs) who have congenital symptoms including hypotonia, respiratory failure and significant cognitive impairment. This congenital phenotype is not only more severe than adult onset DM1, it has some qualitatively different features (Harper, 1975; Reardon et al., 1993). Notably, the muscle pathology in congenital DM1 more closely resembles a developmental or congenital myopathy (as opposed to a dystrophy) and the cognitive defects are much more profound. Importantly, this congenital phenotype is not present in patients with myotonic dystrophy type II, despite very large CCTG repeat expansions in a different gene, *CNBP*, which similarly leads to nuclear RNA foci colocalized with MBNL (Liquori et al., 2001).

Over the past 20 years since the identification of the causative gene in DM1, significant progress has been made in understanding the pathogenic mechanisms involved in the adult onset form of this disease, including the generation of numerous animal model systems in mouse, *C. elegans* and *Drosophila* (Mankodi et al., 2000; de Haro et al., 2006; Mahadevan et al., 2006; Orengo et al., 2008). However, less success has been achieved in attempts to model the congenital form of this disease, where even large expansions in mice have not recapitulated key features of the human disorder (Gomes-Pereira et al., 2007). Of note, few whole-animal based studies have focused on the effects of (CUG) expansion mRNA in early development. Limited studies in human fetuses and more recently in human embryonic stem cell-derived neurons suggest that abnormalities in early development might be important in congenital DM1 phenotypes (Furling et al., 2003; Marteyn et al., 2011). Indeed, some investigators have proposed that very large repeat expansions might trigger temporally aberrant expression of the expanded repeat during early development as a result of local chromatin changes induced by the repeat expansion (Filippova et al., 2001; Cho et al., 2005; Cho and Tapscott, 2007). In this model, both the size of the repeat and the timing of its expression during early development contribute to toxicity.

To explore the impact of CUG RNA expression during early development, we turned to the zebrafish as a model system. Zebrafish offer significant advantages over other model systems because of their rapid development, simple motor phenotypes and the ability to directly introduce RNA, DNA or morpholino constructs at the single cell stage. In the past few years, zebrafish have proven to be powerful systems for understanding the mechanistic underpinnings of neuromuscular disease as well as useful tools for early therapeutic drug screens (Guyon et al., 2003; Dowling et al., 2009; Dowling et al., 2010; Telfer et al., 2010; Gupta et al., 2011; Kawahara et al., 2011).

**TRANSLATIONAL IMPACT**

**Clinical issue**

Myotonic dystrophy type I (DM1) is the third most common muscular dystrophy worldwide, affecting thousands of people. It results from expression of a toxic CUG repeat-containing mRNA that binds to and sequesters specific RNA-binding proteins including muscleblind, which is involved in splicing regulation. Very large expansions of this CUG repeat lead to a congenital form of DM1 characterized by intellectual disability and severe weakness; features that are not seen in adults with the disease. Despite significant advances in our understanding of the genetics and biology underlying this disorder, there are still no effective treatments for DM1. An important unanswered question in the field is what impact the DM1 mutation has during early developmental processes. There is also a pressing need for *in vivo* model systems that allow for rapid therapeutics screening of compounds targeted at blocking CUG repeat-elicited toxicity.

**Results**

This paper describes a novel zebrafish model of DM1 based on injection of mRNA that contains an expanded CUG repeat. This model displays a number of early developmental abnormalities including morphologic, motoric and transcriptional abnormalities within the first 24–48 hours after fertilization. These findings recapitulate some, but not all, of the features observed in adult models of DM1 and demonstrate that the CUG RNA repeat can be toxic despite having limited impact on mRNA splicing. Importantly, the authors demonstrate that these abnormalities are all correctable by coexpression of the RNA-binding protein muscleblind.

**Implications and future directions**

These data provide evidence that CUG repeat RNA can interfere with early developmental processes in zebrafish, offering insights into the pathogenesis of myotonic dystrophy. In particular, these findings have implications for understanding the congenital form of the disease, which is clinically very different to the adult counterpart. The novel model system described here provides a valuable platform for small-molecule screening aimed at the development of new therapies for DM1.

Here we describe a transient DM1 zebrafish model using microinjection, at the single-cell stage, of *in vitro* transcribed mRNA encoding GFP fused to the *DMPK* 3′UTR containing 91 CUG repeats. *GFP(CUG)_91_* mRNA injection leads to alterations in morphology, behavior and the transcriptional profile during early development. Surprisingly, these changes are not accompanied by alterations in four known MBNL splicing targets. Despite this, coexpression of *mbnl2* with *GFP(CUG)_91_* RNA leads to correction of the motor, morphologic and transcriptomic defects. Taken together, this new model system confirms the potential impact of toxic CUG repeat-containing mRNA on early developmental processes and provides a valuable new tool in the armamentarium of research on myotonic dystrophy.

## RESULTS

### Zebrafish embryos injected with *GFP(CUG)_91_* mRNA demonstrate increased early mortality and morphological defects

**Fig. 1:**
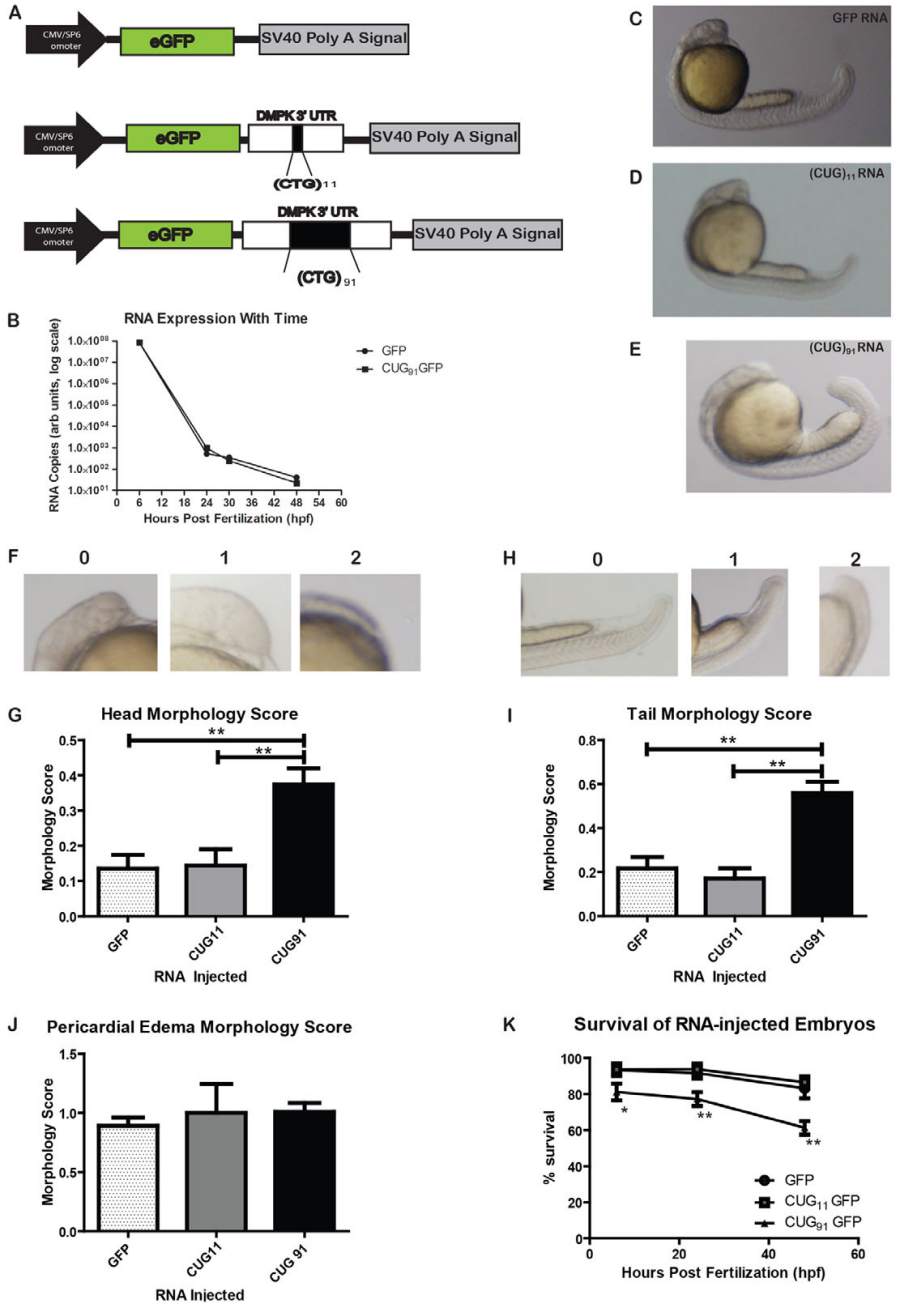
**Expanded CUG repeat RNA injection elicits morphological abnormalities in zebrafish embryos.** (A) Schematic of constructs used to generate *in vitro* transcribed mRNA. (B) Injected RNA stability is similar for *GFP* and *GFP(CUG)_91_* mRNAs, as assessed by qRT-PCR. Expression is normalized to actin mRNA from the same samples at all time-points; *n*=10 fish per group at each time point. (C-E) Representative embryos at 24 hpf injected with *GFP* mRNA (C), *GFP(CUG)_11_* mRNA (D) or *GFP(CUG)_91_* mRNA (E). (F) Abnormal head phenotypes observed in some *GFP(CUG)_91_* mRNA-injected embryos at 24 hpf. 0, normal; 1, mild abnormalities; 2, severe abnormalities. (G) Blinded quantification of the abnormal head phenotypes across groups. (H) Abnormal tail and body shape phenotypes observed in some *GFP(CUG)_91_* mRNA-injected embryos at 24 hpf. (I) Blinded quantification of the abnormal tail phenotypes across groups. (J) Blinded quantification of pericardial edema across groups. (K) *GFP(CUG)_91_* mRNA-injected embryos have increased mortality at 24 and 48 hpf compared with *GFP* or *GFP(CUG)_11_* mRNA-injected embryos. Graph shows survival of embryos injected with the indicated RNAs over 48 hours. Data from G, I, J and K represent *n*>200 embryos per group and at least five independent experiments. *P<0.05, **P<0.001. For morphological assessments, this represents the Dunn post-hoc multiple comparison test after confirmation of significant differences by the Kruskal-Wallis one-way ANOVA. For K, this represents a chi-squared test.

To investigate the impact of expression of mRNA containing CUG repeats during early development, we injected wild-type (AB) zebrafish embryos at the 1–2 cell stage with mRNA encoding GFP alone or GFP with the 3′UTR of *DMPK* and (CUG)_11_ repeats or (CUG)_91_ repeats (Fig. 1A). The stability of mRNA was similar for all constructs, with levels highest in the first 6 hours post fertilization (hpf), then decreasing at 24–48 hpf and essentially undetectable by 72 hpf (Fig. 1B). Injection of *GFP(CUG)_91_* mRNA, but not *GFP(CUG)_11_* mRNA, caused markedly increased mortality of embryos at 24 and 48 hpf compared with *GFP* mRNA at equivalent concentrations (Fig. 1K).

At 24 hpf, the majority of embryos injected with *GFP(CUG)_91_* mRNA exhibited morphological abnormalities (Fig. 1E compared with 1C,D). These included mal-development or delayed development of the head and forebrain structures, and occasionally (~5%) resulting in an anencephalic phenotype (Fig. 1F). Similarly, there was abnormal development of the tail and musculoskeletal system, with foreshortening or curvature of the tail observed in the majority of embryos (Fig. 1H). Some embryos were also noted to have increased pericardial edema (images not shown). To more quantitatively assess the severity of the abnormalities observed, a morphological scoring system was established for measuring these phenotypes (see Materials and Methods for details). A rater blinded to the injected RNA group evaluated >300 embryos from each group across six independent experiments. There was a significant incidence of both head and tail developmental abnormalities in *GFP(CUG)_91_* mRNA-injected fish compared with *GFP(CUG)_11_* and *GFP* mRNA-injected embryos (Fig. 1G,I). All three groups had similar rates of pericardial edema, suggesting that this aspect of the phenotype was not specific to expanded CUG RNA (Fig. 1J). Despite these gross morphologic abnormalities, neither *GFP(CUG)_91_* nor *GFP(CUG)_11_* mRNA-injected fish exhibited alterations in their muscle architecture at 48 hpf compared with *GFP* mRNA-injected embryos, with preservation of nuclear position and normal formation of sarcolemmal structures (contractile units and triads), as examined by semithin section and transmission electron microscopy (TEM) image analysis (supplementary material Fig. S1). These embryos also exhibit normal myofiber integrity, as measured by birefringence at 48 hpf (supplementary material Fig. S1) (Gibbs et al., 2013).

### Zebrafish embryos injected with *GFP(CUG)_91_*mRNA display developmental motor dysfunction

**Fig. 2:**
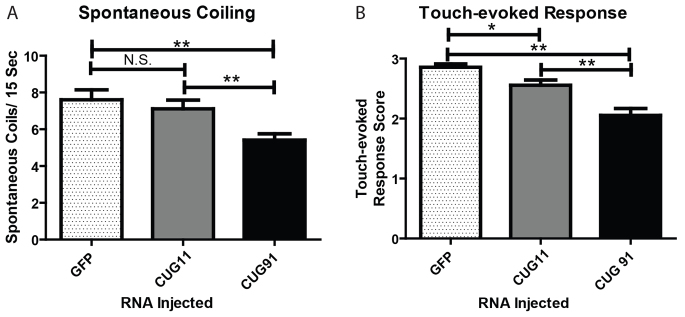
***GFP(CUG)_91_* mRNA-injected embryos display motor dysfunction.** (A) Spontaneous coiling behavior at 24 hpf in embryos injected with the indicated RNAs. Graph shows the average number of spontaneous coiling movements per 15 seconds. (B) Touch-evoked swim escape response at 48 hpf in embryos injected with the indicated RNAs. For both A and B, *n*>100 for each group from five independent experiments, *P<0.05, **P<0.001.

Zebrafish models of neuromuscular disorders often exhibit abnormalities in basic motor behaviors during early development (Dowling et al., 2010; Telfer et al., 2010). The first observable indication of skeletal muscle activity is spontaneous coiling, i.e. the alternating contraction of trunk and tail that begins at 17 hpf, peaks at 19 hpf and then decreases over the next 8 hours (Drapeau et al., 2002). We monitored and quantified spontaneous coiling at 24 hpf. There was a significant decrease in the rate of spontaneous coiling events in *GFP(CUG)_91_* mRNA-injected embryos compared with *GFP(CUG)_11_* or *GFP* mRNA-injected embryos (*GFP* mRNA, 7.61±0.54/15 seconds; *GFP(CUG)_11_* mRNA, 7.11±0.47/15 seconds; *GFP(CUG)_91_* mRNA, 5.40±0.34/15 seconds; *n*>50/group; *P*<0.001 for *GFP* versus *GFP(CUG)_91_* mRNAs; Fig. 2A). A second observable skeletal muscle-dependent phenotype in zebrafish embryos is the touch-evoked swim response. Touch-evoked behaviors begin as rapid alternating contractions of the trunk and tail in response to touch and later (at 27 hpf) incorporate swimming to these rapid alternating contractions, which propel the embryos forward. Touch-evoked escape behaviors grow in strength such that, at 48 hpf, embryos are capable of generating bouts of swimming lasting several seconds. We therefore assessed the touch-evoked escape response at 48 hpf in our RNA-injected embryos. There was significant impairment in the touch-evoked swim response in *GFP(CUG)_91_* mRNA-injected embryos at 48 hpf compared with *GFP* mRNA-injected embryos using an established objective scoring system [*GFP* mRNA, 2.86±0.05; *GFP(CUG)_91_* mRNA, 2.06±0.11; *n*>50/group; *P*<0.001; Fig. 2B] (Dowling et al., 2009). There was a small effect on touch-evoked escape response in *GFP(CUG)_11_* mRNA-injected embryos compared with *GFP* mRNA-injected controls [*GFP(CUG)_11_* mRNA, 2.56±0.08; *n*>50; *P*<0.05 versus *GFP* mRNA; Fig. 2B], which was the only statistically significant difference observed between controls and *GFP(CUG)_11_* mRNA-injected embryos.

### RNA foci zebrafish embryos injected with *GFP(CUG)_91_* mRNA and DNA

**Fig. 3:**
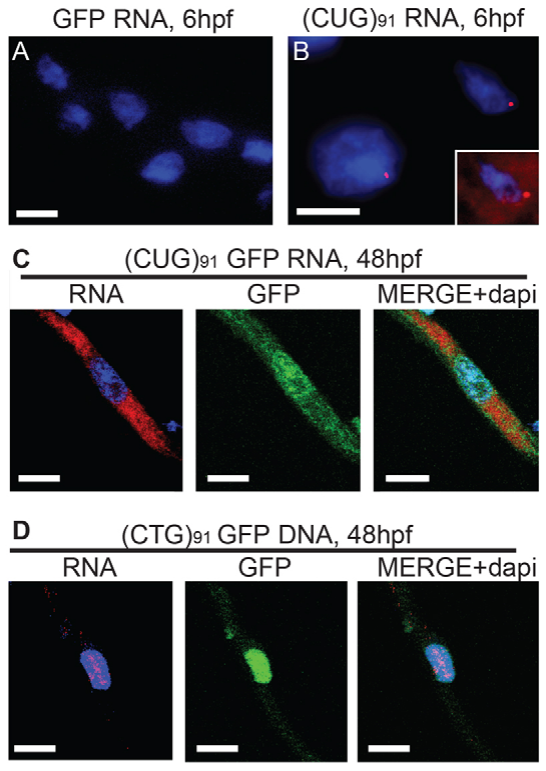
**RNA foci formation in *GFP(CUG)_91_* mRNA-injected embryos.** (A) *In situ* hybridization using a Cy5–2-*O*-methyl (CAG)_5_ RNA probe in *GFP* mRNA-injected embryos at 6 hpf. There was significant GFP visible in all the cells (not shown) but no foci. (B) *In situ* hybridization using a Cy5–2-*O*-methyl (CAG)_5_ RNA probe in *GFP(CUG)_91_* mRNA-injected embryos at 6 hpf. Foci were readily visible in many nuclei. In addition, there was significant diffuse nuclear and cytoplasmic Cy5 signal above that seen in *GFP* mRNA-injected embryos (inset). (C) Dissociated myofibers from *GFP(CUG)_91_* mRNA-injected embryos isolated at 48 hpf. Nuclear RNA foci were rarely seen, but RNA was still present diffusely in the cytoplasm and (to a lesser degree) the nucleus. (D) Dissociated myofibers from *GFP(CUG)_91_* DNA-injected embryos isolated at 48 hpf. Nuclear RNA foci were readily visible in GFP-positive myofibers, with very little visible diffusely in the nucleus or cytoplasm. Scale bars: 20 μm.

RNA foci are a pathological hallmark of DM1 in affected tissues and accumulate predominantly in terminally differentiated cells. The formation of these foci is thought to occur peri-transcriptionally, with accumulation of the foci and associated RNA-binding proteins (such as MBNL) into pre-splicosomal SC35-positive complexes (Smith et al., 2007). However, exogenously introduced *in vitro* transcribed RNA containing an expanded CUG repeat probably gains access to the nucleus during cellular division and might not interact directly with the pre-splicosomal complex. We therefore evaluated the localization of CUG RNA and whether foci form in this model. Using 2-*O*-methyl Cy5-labeled (CAG)_6_ oligonucleotide probes, we performed *in situ* hybridization in *GFP* mRNA- or *GFP(CUG)_91_* mRNA-injected zebrafish at 6, 24 and 48 hpf. At 6 hpf, nuclear RNA foci were seen in GFP-positive cells from both *GFP(CUG)_11_* and *GFP(CUG)_91_* mRNA-injected embryos but not *GFP* mRNA-injected embryos (Fig. 3A,B; supplementary material Fig. S2). In addition, in *GFP(CUG)_91_* mRNA-injected embryos, CUG RNA was detected diffusely in the nucleus and cytoplasm at 6 hpf, significantly above the background level seen in embryos injected with *GFP* mRNA (Fig. 3B, inset). At 24 or 48 hpf, no nuclear foci were seen in *GFP(CUG)_91_* mRNA-injected embryos, but cytoplasmic and nuclear RNA was still visible in dissociated myofibers (Fig. 3C; supplementary material Fig. S2). In contrast, in *GFP(CUG)_91_* DNA-injected embryos, which exhibit chimeric GFP expression in muscle and other tissues, RNA foci were readily observed in GFP-positive dissociated myofibers derived at 48 hpf (Fig. 3C). These data suggest that nuclear CUG RNA foci can form with delivery of exogenously transcribed *GFP(CUG)_91_* mRNA. However, the formation of these foci might be less efficient and, once formed, they could be less stable than foci generated by CUG repeats transcribed *in vivo.*

### RNA splicing in zebrafish embryos injected with *GFP(CUG)_91_* mRNA

**Fig. 4:**
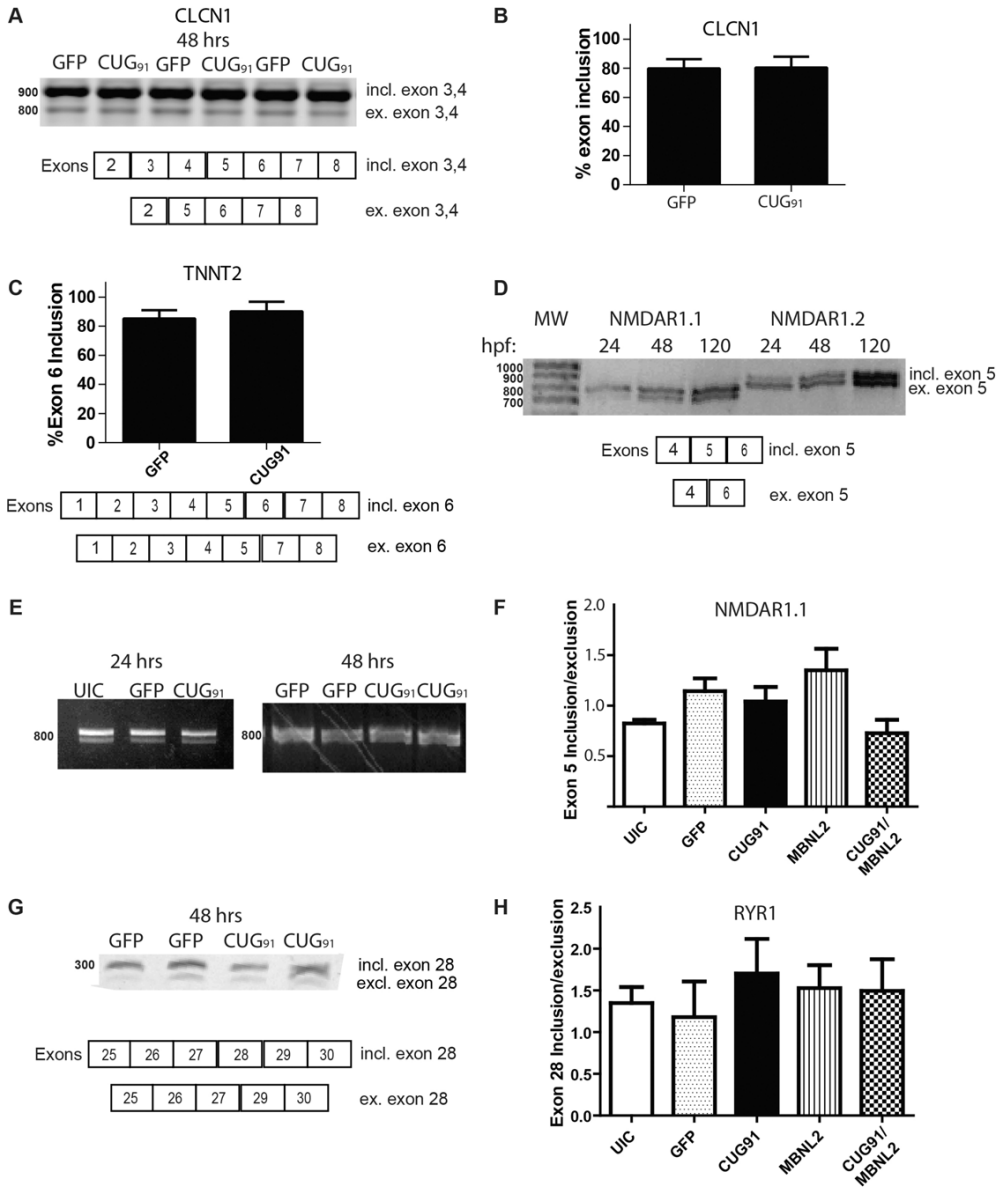
**Splicing in *GFP(CUG)_91_* mRNA-injected embryos.** (A) CLCN1 splicing patterns in three independent sets of *GFP* and *GFP(CUG)_91_* mRNA-injected embryos at 48 hpf. The two major isoforms observed at 800 and 900 nucleotides (nt) were sequence confirmed and reflect the inclusion or exclusion of exons 3 and 4. (B) Quantification of exon 3 and 4 retention in *GFP* and *GFP(CUG)_91_* mRNA-injected embryos at 48 hpf. (C) Quantification of exon 6 retention in TNNT2 in *GFP* and *GFP(CUG)_91_* mRNA-injected embryos at 48 hpf. (D) Both *NMDAR1.1* and *NMDAR1.2* mRNA undergo age-dependent splicing alterations in relation to inclusion or exclusion of exon 5. PCR across the exon 4–6 junction reveals increased exclusion of exon 5 from 24 to 120 hours in uninjected zebrafish embryos. In contrast, NMDAR1.2 shows increased inclusion of exon 5 over the same timeframe. (E) Injection of *GFP* mRNA or *GFP(CUG)_91_* mRNA does not alter splicing patterns of NMDAR1.1 at 24 or 48 hpf. (F) Quantification of NMDAR1.1 splicing at 48 hpf in embryos injected with the indicated RNAs. (G) Splicing of RYR1 at 48 hpf in *GFP* or *GFP(CUG)_91_* mRNA-injected embryos. The higher molecular weight band represents retention of exon 28 whereas the lower band reflects its exclusion. (H) Quantification of RYR1 intron/exon ratio at 24 hpf using qRT-PCR with primers located either within or outside of exon 28. For B, C, F and H significant differences were observed in three independent experiments across any of the groups by one-way ANOVA.

In mouse models of myotonic dystrophy and in patient tissues, expanded CUG RNA is associated with the mis-splicing of numerous transcripts. These aberrant splicing events contribute to clinical symptoms, including myotonia (Philips et al., 1998; Mankodi et al., 2002; Jiang et al., 2004; Wheeler et al., 2007; Orengo et al., 2008; Osborne et al., 2009). The majority of these splicing abnormalities involve the retention of fetal isoforms of transcripts into adulthood. These mis-splicing events correlate with a loss of MBNL function elicited by the sequestration of MBNL by CUG repeat RNA (Orengo et al., 2008; Osborne et al., 2009; Du et al., 2010). In zebrafish, knocking down expression of *mbnl2* mRNA by morpholino injection also leads to mis-splicing of transcripts, including *tnnt2* and *clcn1*, at 51 hpf (Machuca-Tzili et al., 2011). We therefore evaluated the splicing of these two transcripts in *GFP* mRNA-injected and *GFP(CUG)_91_* mRNA-injected embryos at 48 hpf. PCR amplification of *clcn1* variants in control zebrafish at 48 hpf revealed two distinct splice variants, correlating with inclusion or exclusion of exons 3 and 4 in zebrafish *clcn1*, which was confirmed by sequencing (Fig. 4A and not shown). When the expression of these splice isoforms was compared between *GFP* and *GFP(CUG)_91_* mRNA-injected embryos, there were no significant differences at either 24 or 48 hpf (Fig. 4A,B and data not shown). The normal splicing observed in these RNA-injected embryos was not reflective of an insensitivity for detection, as mosaic *GFP(CUG)_91_* DNA-injected embryos exhibited robust shifts in *clcn1* splicing at 24 and 48 hpf (supplementary material Fig. S3 and data not shown). Similarly, *GFP* and *GFP(CUG)_91_* mRNA-injected embryos exhibited a similar ratio of *tnnt2* splice isoforms at 48 hpf (Fig. 4C).

Given that we had expected to see alterations in MBNL target transcript splicing, we sought out additional potentially dysregulated splicing events in injected zebrafish. To accomplish this, we first identified splicing events that were known to be abnormal in mouse models or patient-derived tissues and that were conserved in zebrafish. For example, the NMDAR1 receptor is known to be mis-spliced in DM1 human brain tissue (Jiang et al., 2004). This missplicing leads to an increased retention of exon 5, which influences the distribution of the channel (Durand et al., 1993; Pal et al., 2003). As previously reported (Cox et al., 2005), the *NMDAR1.1* and *NMDAR1.2* mRNA splicing dynamics of exon 5 are conserved in zebrafish and undergo a transition during early development (Fig. 4D). However, we saw no differences in the splicing of NMDAR1.1 at 24 or 48 hpf between *GFP* and *GFP(CUG)_91_* mRNA-injected embryos either by gel analysis or by isoform specific qRT-PCR (Fig. 4E,F).

We next analyzed alternative splicing of *ryr1b* mRNA. Expression of *ryr1b* and splicing are developmentally regulated in zebrafish (Wu et al., 2011) and RYR1 splicing is abnormal in DM1 patients and in CUG RNA-expressing mice (Kimura et al., 2005). Analysis of the intron-exon borders in zebrafish showed sequence conservation between mammals and zebrafish for the *ryr1α* exon (exon 28 in zebrafish) that is mis-spliced in DM1 patients. Using primers outside of this exon, we first determined the relative abundance of the two splice variants. At 24 and 48 hours, the majority of transcripts contain this exon regardless of the RNA injected (Fig. 4G). Using primers located either within or outside of exon 28, we confirmed this ratio of expression, but saw no differences in the ratios of splice isoforms between *GFP* and *GFP(CUG)_91_* mRNA-injected embryos at 48 hpf by qRT-PCR (Fig. 4H).

### Zebrafish embryos injected with *GFP(CUG)_91_* mRNA have altered transcriptional profiles reflecting delayed development

**Fig. 5:**
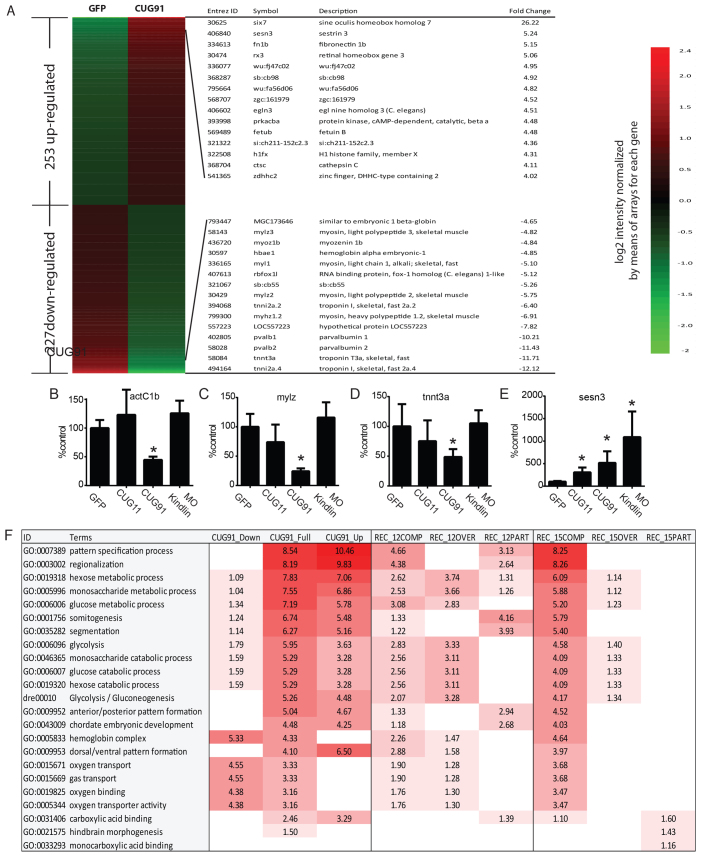
**Transcriptome abnormalities in *GFP(CUG)_91_* mRNA-injected embryos.** (A) Heat map showing genes up- or downregulated at least twofold between *GFP* and *GFP(CUG)_91_* mRNA-injected embryos at 24 hpf. 480 differentially expressed genes (DEGs) were dysregulated in *GFP(CUG)_91_* mRNA-injected fish, included 253 genes upregulated in (CUG)_91_ and 227 genes downregulated in (CUG)_91_. Their expression levels, represented by the intensities on the microarrays, were log2 transformed and normalized to the mean value of each gene across GFP and (CUG)91 arrays. The 15 genes with the most substantial changes by *GFP(CUG)_91_* mRNA treatment are listed for each group. (B) qRT-PCR to cardiac muscle α-actin 1b (actC1b) at 24 hpf from embryos injected with the indicated transcripts. MO kindlin is an antisense morpholino knockdown of kindlin-1 used as a control for nonspecific toxicity. (C) qRT-PCR to myosin light polypeptide 2 (mylz2) at 24 hpf from embryos injected with the indicated transcripts. (D) qRT-PCR to troponin T3a (tnnt3a) at 24 hpf from embryos injected with the indicated transcripts. (E) qRT-PCR against sestrin 3 (sesn3) in mRNA-injected embryos at 24 hpf. (F) Functional enrichment analyses were performed to identify the most significantly over-represented biological functions or pathways among the genes dysregulated by *GFP(CUG)_91_* mRNA. Down: significantly downregulated genes by CUG91. Up: significantly upregulated genes by CUG91. Full: all significantly dysregulated genes by CUG91. REC: recovery by *mbnl2* injection. COMP: complete recovery with *mbnl2* co-injection. OVER: overcompensation of changes with *mbnl2* co-injection. PART: partial recovery by *mbnl2* co-injection. The values correspond to ‘–log10(EASE score)’ for each functional term, calculated by the Database for Annotation, Visualization and Integrated Discovery (DAVID). EASE score corresponds to the *P*-value calculated by a modified Fisher’s exact test. A value of 2 corresponds to a *P*-value of 0.01. The color gradient is based on the *P*-value, with lower *P*-values being darker. Muscle developmental and energy metabolism pathways were over-represented in the altered transcripts. *P<0.05 versus *GFP* mRNA-injected embryos on Fisher’s least significant difference test.

Mouse models of DM1 and analysis of human DM1 muscle tissues reveal significant transcriptional dysregulation (Osborne et al., 2009; Du et al., 2010). Interestingly, 60–70% of these transcriptional changes are recapitulated in MBNL1 knockout mice (Osborne et al., 2009). Given the lack of abnormal splicing in candidate transcripts during early development, we evaluated transcript expression by microarray in zebrafish embryos at 24 hpf. Compared with fish injected with *GFP* mRNA alone, there was a greater than twofold alteration in the expression of 480 genes, with roughly an equal number of transcripts up and downregulated (Fig. 5A; supplementary material Tables S1, S2). Interestingly, mRNA levels of a number of crucial muscle transcripts, including troponin I and T isoforms, and multiple myosin heavy and light chain isoforms, were among the most downregulated genes (Fig. 5A–D; supplementary material Table S2). In contrast, sestrin 3, which is a marker of inflammation and oxidative stress and which has been implicated in some neurological disorders, was the second most highly elevated transcript (Fig. 5A,E; supplementary material Table S2). There were no significant changes in *mbnl2* (86.3%), *celf1* (100.2%), *celf2* (97.3%) or *celf3* (93.5%) transcript quantity associated with CUG repeat expression.

The vast majority (>95%) of the dysregulated transcripts identified were not previously found in microarray analyses performed in mouse models of myotonic dystrophy or from adult-patient derived tissues (supplementary material Fig. S4) (Osborne et al., 2009; Du et al., 2010; Wang et al., 2012). We selectively validated alterations in a set of these transcripts, including downregulation of the muscle-specific transcripts myosin light chain 2 and *tnnt3a* and upregulation of the stress-activated protein sestrin 3 (Fig. 5B–E). To determine whether the changes were specific to expression of the expanded CUG repeat, we also assessed whether similar transcriptional changes were induced by morpholino knockdown of kindlin-2, which is an integrin-associated protein found in cardiac and striatal muscle (Dowling et al., 2008). The kindlin-2 morphants have severe developmental abnormalities in their hearts, muscle and central nervous system. Not surprisingly, some transcriptional changes were shared between (CUG)_91_ and kindlin-2 morphants (for example, sestrin 3) (Fig. 5E). However, the downregulation of muscle-specific transcripts including *act1b*, *mlz2* and *tnnt3a* was not recapitulated in the kindlin-2 embryos, suggesting that these effects were more specific to the CUG repeat RNA (Fig. 5B–D). Similarly, *GFP(CUG)_11_* mRNA-injected embryos exhibited few significant changes from *GFP* mRNA-injected controls (Fig. 5B–E), suggesting that the effects were dependent on the repeat length.

Gene ontology (GO) and Kyoto Encyclopedia of Genes and Genomes (KEGG) analysis identified a number of specific areas of dysregulation, including a global downregulation of muscle developmental pathways, downregulation of hemoglobin and gas transport and an upregulation of energy metabolism-associated transcripts (Fig. 5F). These changes might reflect a delay in muscle maturation induced by the CUG repeat RNA.

### Coexpression of MBNL2 suppresses *GFP(CUG)_91_*mRNA-mediated toxicity

The sequestration of MBNL proteins by CUG RNA repeats is established as an important component of the pathogenesis in adult DM1. In multiple model systems, including *Drosophila* and mice expressing CUG repeats, coexpression of MBNL suppresses CUG-mediated toxicity (de Haro et al., 2006; Kanadia et al., 2006). In addition, MBNL1 and MBNL2 knockout mice exhibit a number of cardinal features also seen in mice expressing CUG RNA and in DM1 patients (Kanadia et al., 2003a; Charizanis et al., 2012). These studies are generally interpreted as indicating that replacement of sequestered MBNL is sufficient to correct many cardinal disease phenotypes in DM1. Because the morphologic phenotype we observe with *GFP(CUG)_91_* mRNA injection is similar to that reported for *mbnl2* morpholino-injected embryos (Machuca-Tzili et al., 2011), we investigated whether coexpression of zebrafish *mbnl2* with *GFP(CUG)_91_* mRNA would mitigate the phenotype.

**Fig. 6:**
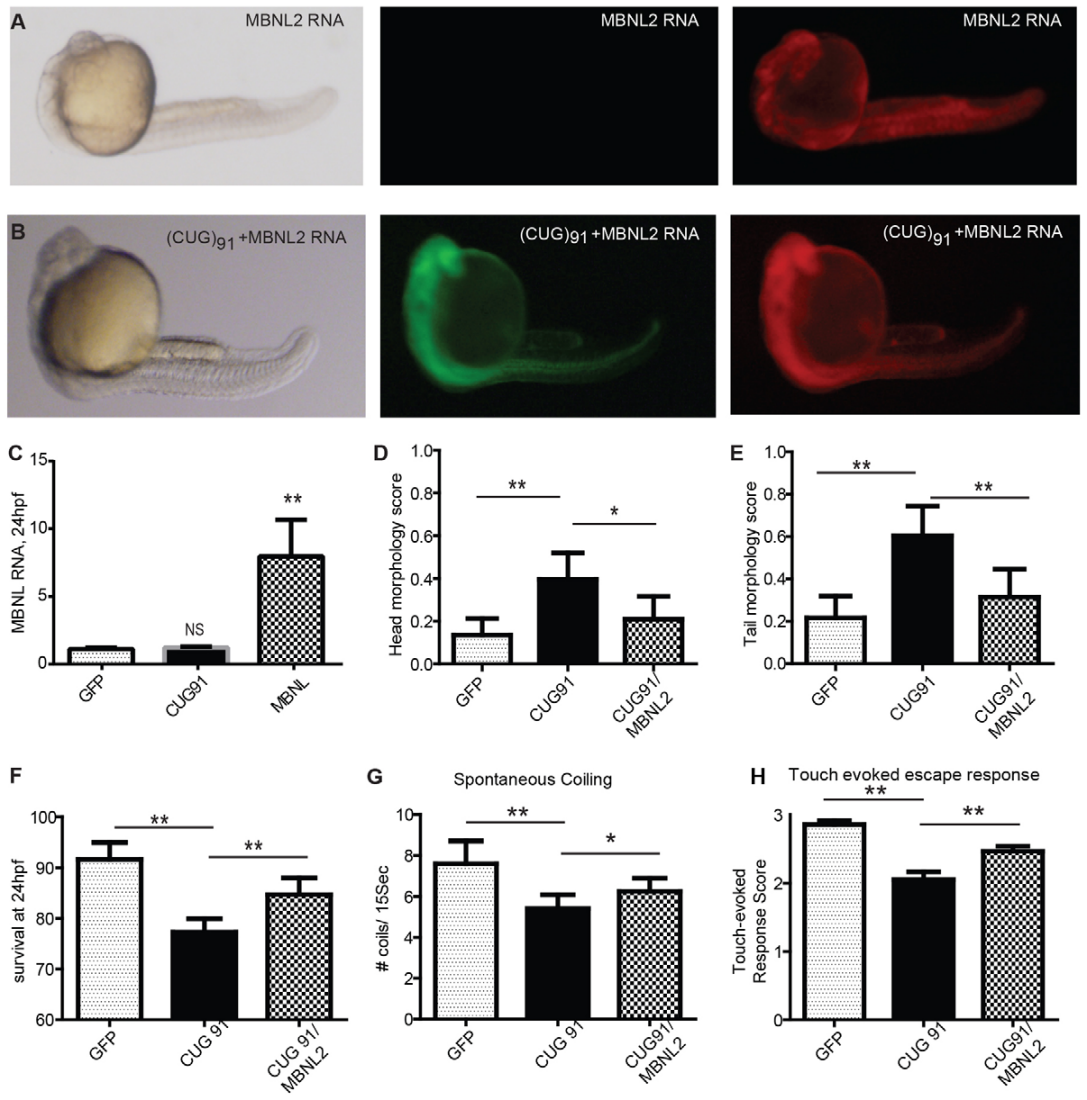
**Coexpression of MBNL2 suppresses *GFP(CUG)_91_* mRNA-induced phenotypes.** (A) Representative images at 24 hpf of an embryo injected with mRNA encoding MBNL2-mCherry. (B) Representative images at 24 hpf of an embryo co-injected with mRNAs encoding MBNL2-mCherry and GFP(CUG)_91_. (C) Quantification of *mbnl2* mRNA by qRT-PCR in *GFP* mRNA, *GFP(CUG)_91_* mRNA and MBNL2-mCherry-injected embryos at 24 hpf. (D) Blinded quantification of the abnormal head phenotypes in embryos injected with the indicated RNAs. (E) Blinded quantification of the abnormal tail phenotypes in embryos injected with the indicated RNAs. (F) Survival at 24 hpf in embryos injected with the indicated RNAs. (G) Spontaneous coiling behavior at 24 hpf in embryos injected with the indicated RNAs. (H) Touch-evoked swim escape response at 48 hpf in embryos injected with the indicated RNAs. For D-H, more than 100 embryos per group were evaluated in at least three independent experiments. Equal amounts of *GFP(CUG)_91_* mRNA were injected in the presence or absence of *mbnl2* mRNA. *P<0.05, **P<0.001 on two-tailed unpaired *t*-test except for survival data, which was analyzed using a chi-squared test.

Injection of *mbnl2* mRNA by itself at 500 pg/embryo had mild phenotypic effects compared with similar concentrations of *GFP* mRNA injected alone (data not shown). We therefore used a lower concentration of 50 pg, which had no significant effect on the morphological phenotype (Fig. 6A,C). However, co-injection of *mbnl2* mRNA with *GFP(CUG)_91_* mRNA strongly suppressed the CUG RNA-induced morphologic phenotypes observed at 24 hpf (Fig. 6B). The correction of these morphological abnormalities was assessed blinded to the RNA injected into the embryos. A significant correction was observed for both the head and tail morphological abnormalities (Fig. 6D,E). Consistent with this, survival at 24 hpf was enhanced by co-injection of *mbnl2* mRNA with *GFP(CUG)_91_* mRNA compared with injection of *GFP(CUG)_91_* mRNA alone (Fig. 6F).

We next evaluated whether coexpression of *mbnl2* affected the motor phenotypes observed at 24 or 48 hpf in *GFP(CUG)_91_* mRNA-injected embryos. As with the morphological phenotypes, *mbnl2* injection alone did not have a significant impact on either spontaneous coiling frequency at 24 hpf or on the touch-evoked swim response at 48 hpf. However, coexpression of *mbnl2* mRNA with *GFP(CUG)_91_* mRNA partially corrected both of these motor phenotypes (Fig. 6G,H).

**Fig. 7:**
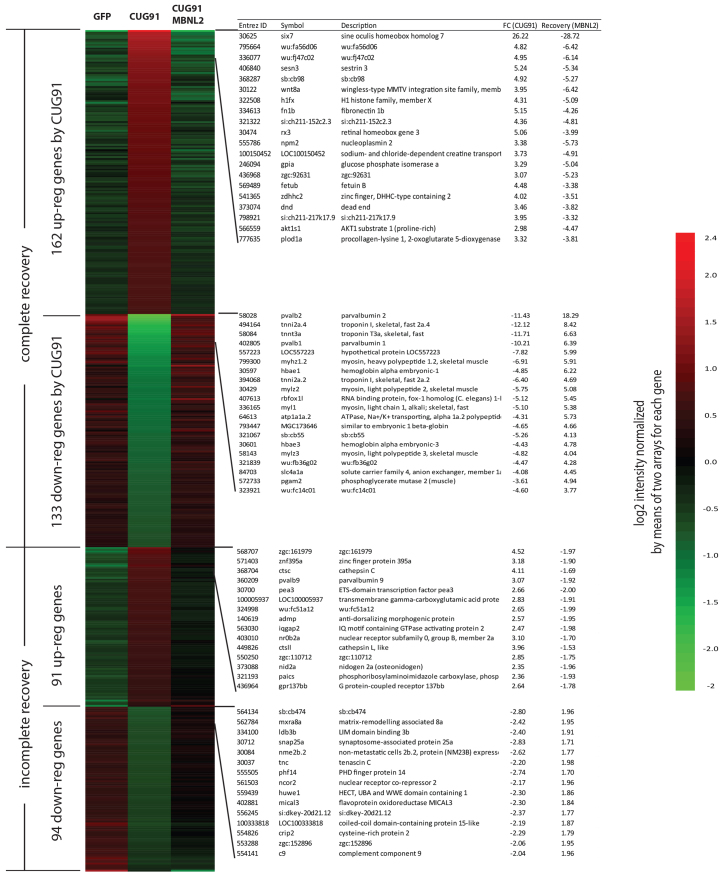
**Coexpression of MBNL2 suppresses *GFP(CUG)*_91_ mRNA-induced transcriptional abnormalities.** (A) Comparison of transcript changes at 24 hpf of embryos injected with *GFP* mRNA, *GFP(CUG)_91_* mRNA or co-injected with *GFP(CUG)_9_*_1_ and *mbnl2* mRNAs. There was very little overlap in transcripts altered by overexpression of *mbnl2* and *GFP(CUG)_91_* mRNAs. (B) Heat map represents changes in transcript expression between embryos at 24 hpf injected with the indicated RNAs. Correction was considered complete if the transcript expression was within 1.5-fold of that seen with *GFP* mRNA-injected embryos and incomplete if between 1.5- and 2.0-fold of that seen with *GFP* mRNA-injected embryos. Only three transcripts were not at least partially corrected by co-injection of MBNL2-encoding mRNA.

Recent studies of transcriptional expression changes associated with CUG RNA expression suggest that many of the alterations can be recapitulated by knocking out *mbnl1* (Osborne et al., 2009). We therefore evaluated the impact of *mbnl2* expression on GFP(CUG)_91_-associated transcriptional abnormalities. Injection of *mbnl2* mRNA alone had a limited effect on the transcriptional profile of embryos at 24 hpf compared with embryos injected with *GFP* mRNA (supplementary material Tables S1, S2). Moreover, these changes were largely unrelated to those observed with *GFP(CUG)_91_* mRNA injection (supplementary material Fig. S5). Despite this, coexpression of *mbnl2* mRNA with *GFP(CUG)_91_* mRNA led to a significant correction of the transcriptomic abnormalities observed (Fig. 7). The majority (63%) of transcripts were corrected back to levels comparable to that in embryos injected with *GFP* mRNA alone (within 1.2-fold of the expression levels with *GFP* mRNA alone), with partial correction of most other transcripts. Using a less stringent criteria for a complete recovery (1.5-fold change), ~93% of the deregulated genes were completely recovered by coexpression with *mbnl2* mRNA. Remarkably, only 3 of 488 (0.62%) of the deregulated transcripts were unaltered by coexpression of *mbnl2*.

### Zebrafish injected with *GFP(CUG)_91_* mRNA have normal motor phenotypes as adults

Because CUG RNA degrades by 72 hpf, the injection of CUG RNA into single-cell embryos allows us to assess the long-term effects on muscle and motor development of this transient developmental expression. We therefore analyzed the impact of embryonically injected *GFP* mRNA and *GFP(CUG)_91_* mRNA at 1 and 31 weeks after fertilization, when effectively all the injected RNA has been degraded. In embryos that survived the first 48 hours, there was no significant alteration in survival at 1 week (supplementary material Fig. S6A). To analyze the motor function in these fish, we utilized the Noldus DanioVision Infrared swim tracking apparatus (Dowling et al., 2010; Telfer et al., 2010). At 1 week, there was no notable difference in the average swim speed or total distance swum in a 10 minute period (supplementary material Fig. S6B). Similarly, at 31 weeks post fertilization, adult fish that had been injected with *GFP(CUG)_91_* RNA were similar to uninjected controls in terms of swim speed, average velocity and total distance traveled in 10 minutes (supplementary material Fig. S6C). These findings suggest that transient expression of an expanded CUG repeat as RNA does not preclude eventual normal muscle development and function once the inciting toxic RNA has been removed.

## DISCUSSION

Our results demonstrate that CUG repeat RNA expression can elicit toxicity in zebrafish embryos. This toxicity manifests with limited CUG RNA foci formation, morphologic abnormalities, early behavioral abnormalities and significant transcriptional changes. Coexpression of MBNL2 with *GFP(CUG)_91_* mRNA suppresses the observed morphologic, behavioral and transcriptional alterations, suggesting a role for MBNL in these phenotypes. Lastly, embryos that survive the initial developmental period during which the expanded CUG repeat RNA is present have seemingly normal late development and motor function, as measured by swim speed at 1 and 31 weeks of age. Our results establish zebrafish as a model system for studying DM1 pathogenesis and provide insights into this disorder and into RNA-mediated toxicity.

One goal of this work was to establish a system for studying early developmental effects of CUG repeat RNA-mediated toxicity. Congenital myotonic dystrophy is associated with qualitatively different symptoms and signs from its adult onset counterpart, including mental retardation, cortical migration abnormalities, and respiratory difficulties and diffuse hypotonia with myopathic features on muscle biopsies. These outcomes suggest that CUG repeat expansions might have effects during development. Studies in DM1-derived human embryonic stem cells support this idea, with defects in neurite outgrowth and synapse formation associating with dysregulated gene expression of crucial developmental transcripts (Marteyn et al., 2011). However, attempts to address these developmental defects *in vivo* have been limited by both technical difficulties associated with large CUG repeats and by the lack of significant early developmental phenotypes in mouse models (Gomes-Pereira et al., 2007).

Our data support the concept that early expression of a pathogenic CUG repeat can elicit developmental toxicity *in vivo*. These findings are consistent with previous studies in mouse models, where the context of the repeat and its overall expression level are important determinants of toxicity, independent of repeat size (Sabourin et al., 1997; Amack and Mahadevan, 2001; Filippova et al., 2001; Storbeck et al., 2004; Mahadevan et al., 2006). Our data are consistent with a model in which some aspects of CUG repeat-associated developmental toxicity result from when the repeat RNA is expressed rather than the size of the CUG repeat expansion alone. However, because of technical limitations, we have not yet been able to successfully generate large CUG repeat-containing mRNAs by *in vitro* transcription to test this hypothesis empirically.

Aspects of the CUG RNA phenotype we observed mirror those associated with global knockdown of MBNL2 expression in zebrafish embryos (Machuca-Tzili et al., 2011). Moreover, coexpression of MBNL2 dramatically suppresses many aspects of the CUG mRNA repeat-mediated phenotype. This is consistent with previous studies demonstrating suppression of CUG-related toxicity using adeno-associated virus (AAV)-delivered MBNL into muscle (Kanadia et al., 2006). Unlike these previous studies, in zebrafish the CUG RNA phenotype was not directly associated with MBNL-mediated splicing abnormalities, although our analysis was limited to only a few specific transcripts. This probably reflects an important caveat for this model. Unlike DM1 patients and DM1 mice models, the expanded CUG repeat RNA in zebrafish was introduced exogenously and thus was not transcribed in the nucleus. A component of DM1-mediated toxicity is thought to be elicited by the peri-transcriptional sequestration of CUG RNA and its associated RNA-binding proteins, most notably MBNL, within the nucleus. In zebrafish, this exogenously introduced RNA gains entry into the nucleus and forms foci, but a significant amount of the RNA remains diffuse in the cytoplasm. The cytoplasmic localization of the CUG repeat RNA might trigger a different set of pathogenic cascades than those elicited by large transcribed repeat expansions, which are largely retained in the nucleus. However, this alternative localization also offers unique opportunities to study cytoplasmic triggered CUG repeat effects (including RAN translation) that might contribute to toxicity normally, despite being rare events (Zu et al., 2011).

A second caveat of this work relates to the timing of expression of the CUG RNA and the roles of MBNL in splicing. To date, most studies have focused on how CUG repeat RNA sequesters MBNL, leading to retention of embryonic mRNA isoforms of MBNL target transcripts in differentiated adult tissue. In zebrafish embryos, however, we are focusing on precisely the developmental window when those isoforms are normally favored. Thus, if CUG toxicity depended solely on mis-splicing of MBNL targets, we would predict that CUG RNA might have only mild detrimental effects during early development. Instead, we observe significant effects on transcription, development and behavior during early development that occur in the absence of MBNL2-dependent splicing changes.

At least two non-exclusive possibilities could explain this result. First, as suggested above, CUG RNA could be eliciting toxicity through non-MBNL2-dependent mechanisms. CUG RNA is known to trigger a number of other pathogenic cascades, including activation and overexpression of CELF1, mis-regulation of certain microRNA pathways and activation of the double-stranded RNA-dependent protein kinase PKR (Philips et al., 1998; Tian et al., 2000; Orengo et al., 2008; Rau et al., 2011). Under this scenario, MBNL2-dependent suppression of *GFP(CUG)_91_* mRNA-induced toxicity would be mediated by MBNL2-mediated sequestration of CUG repeat RNA, rather than the other way around as traditionally understood. A second possibility is that MBNL2 has other important functions during early development apart from splicing regulation. MBNL is expressed early in development in zebrafish and other organisms, and it contributes to cytoplasmic mRNA localization (Kanadia et al., 2003b; Adereth et al., 2005; Machuca-Tzili et al., 2011; Wang et al., 2012). In zebrafish embryos, CUG RNA might be specifically interfering with these poorly understood cytoplasmic functions or preventing MBNL2 from performing specific nuclear functions related to transcriptional regulation (Osborne et al., 2009; Charizanis et al., 2012).

In summary, exogenously transcribed CUG repeat RNA disrupts normal muscle and nervous system development in zebrafish. These results provide new insights into DM1 pathogenesis and establish a new model system for DM1 research and therapeutic development.

## MATERIALS AND METHODS

### Constructs and *in vitro* transcription

GFP(CUG)_11_ and GFP(CUG)_91_ constructs were derived by sub-cloning out from previously described GFP(CUG)_11_ and GFP(CUG)_100_ constructs (Storbeck et al., 2004) into an pCS2 vector. During cloning of the construct there was contraction from 100 to 91 uninterrupted CTGs. MBNL2-pCS2 was obtained as a kind gift from David Brook. mCherry was PCR cloned in frame into the C-terminal of MBNL2, removing the MBNL2 stop codon. *In vitro* transcription was carried out using a mMessage mMachine kit (Ambion) according to the manufacturer’s protocol. RNA quality and size were confirmed by acrylamide gel electrophoresis and concentration was determined using a Nanodrop spectrophotometer.

### RNA injections

Embryos were isolated after paired mating of AB zebrafish (zFIN, Eugene, OR) and injected at the one- to two-cell stage using a Drummond Nanoject. Diluted RNA (4.6 nl each) was injected at a concentration of 100 ng/μl for all constructs unless otherwise specified (approximate total amount of RNA injected per embryo was 0.46 ng).

### Assays of motor function

Spontaneous coiling and touch-evoked escape responses were measured as previously described (Dowling et al., 2010; Telfer et al., 2010). Briefly, spontaneous coiling was measured at 24 hpf by observing the number of coils in a 15-second period. Touch-evoked escape response was measured at 48 hpf using a scale from 0 to 3: 0, no movement; 1, flicker of movement but no swimming; 2, movement away from probe but with impaired swimming; 3, normal swimming; *n*>200 for each RNA type from a minimum of three different independent experiments. All animal experiments were performed according to the relevant regulatory standards.

### Morphologic analysis and imaging of embryos

Embryos were photographed at 24 and 48 hpf using a Leica MXIII Stereoscope at 2× with a 4× zoom. For fluorescent imaging, an inverted IX-71 fluorescent microscope (Olympus) was utilized with a 4× or 10× zoom. Embryos at 48 hpf were anesthetized using Tricaine prior to photographing. Morphologic analysis was performed blinded to the RNA injected in five independent experiments with *n>200* total embryos per group. Morphologic scales were empirically defined on the basis of observed phenotypes. Briefly embryos were scored based on their external head shape and size and their tail shape and length at 24 hpf. Embryos with normal appearing heads or tails were scored 0. For the head-based scaling system, a score of 1 was applied to heads that were smaller or mis-shaped, with particular attention to the areas anterior to the developing eye. A score of 2 was reserved for severely abnormal head shapes, which included marked microcephaly or anencephaly For the tail-based scaling system, slightly foreshortened tails or curved tails were scored as a 1. Gross mal-development or foreshortening of the tail was scored as a 2.

### Histopathologic analysis

Zebrafish of 24 or 48 hpf were fixed overnight in Karnovsky’s fixative and then processed for embedding in epon by the Microscopy and Imaging Laboratory core facility at the University of Michigan. Semithin sections were stained with toluidine blue and photographed using an Olympus BX-51 upright microscope. Electron microscopy was performed using a Phillips CM-100 transmission electron microscope.

### Birefringence

Analysis of birefringence, which is a measure of myofiber integrity, was performed as previously described (Telfer et al., 2010). Briefly, polarized light was passed through zebrafish embryos and imaged at 200 microsecond exposure. Embryos were positioned relative to the polarized light to produce maximal birefringence illumination.

### *In situ* hybridization

Detection of RNA foci was performed as previously described (Mankodi et al., 2001). Briefly, embryos injected with the indicated RNAs were fixed in 4% paraformaldehyde (PFA) in PBS for 15 minutes at 6, 24 or 48 hpf and then cryosectioned. Embryos were then post-fixed in PFA, washed in PBS, permeabilized with 5% acetone and prehybridized in 2× saline-sodium citrate buffer (SSC) containing 30% formamide. Slides were then incubated at 55°C for 120 minutes in hyb buffer (0.02% BSA, 66 μg/ml torula yeast RNA, 2 mM vanadyl complex, 30% formamide, 2× SSC) with [1 ng/μl Cy5-labeled (CAG)_5_ 2-*O*-methyl RNA probe; IDT]. Slides were washed in hyb buffer without probe, mounted and cover-slipped in Prolong Gold with DAPI (Invitrogen) and imaged by confocal microscopy.

### mRNA splicing analysis

Splicing analysis was carried out in two different ways: Direct comparison of signal intensity on agarose gels and real-time PCR quantification using isoform-specific PCR primers. For all studies, total RNA was isolated at 24 or 48 hpf using Trizol extraction and reverse transcribed (iscript, Biorad) according to the manufacturer’s protocols. For NMDAR1, previously described primer sets were utilized (Cox et al., 2005). PCR to NR1.1 was conducted using 5′F and e6.R primers; NR1.2 PCR utilized 1.2 5′F and 1.2 e7.R primers. For RYR1, the splicing abnormality identified in mouse and human patients was mapped to the zebrafish RYR1 1 gene, with conservation of the basic splice elements. This represented inclusion or exclusion of exon 28 in the zebrafish transcript. PCR to RYR1 across this exon (27 to 29) was conducted using primers RYR1b forward (5′-GTGATGTTCCTCTACACTGTCCTCT-3′) and RYR1b reverse (5′-CGACGCTTCTTCTTCGTCCG-3′). PCR samples were separated by low-melt agarose electrophoresis and visualized by ethidium bromide staining. Images were captured on a VersaDoc imaging system (Bio-Rad) and quantified using ImageJ software (NIH, Bethesda, MD). PCR cycle number (35 cycles for both NR1.1 and 1.2, 32 for RYR1b) for comparison of splice isoforms was confirmed empirically. To more accurately quantify the splice variants of RYR1b, we utilized real-time RT-PCR with primers that crossed the exon-exon boundaries at 27/28 or 27/29, with a shared downstream primer (27/28 splice primer 5′-AGCAAGGCCGGAGATGGA-3′, 27/29 splice primer 5′-TGAGCAAGGGTGGAGGCTC-3′; reverse primer as above). Real-time RT-PCR was performed as described below. Analysis of splice variants TNNT3a and CLCN1 were performed as previously described (Machuca-Tzili et al, 2011).

### Affymetrix microarray analysis

Pooled RNA for each group were amplified, biotin-labeled using Ovation Biotin-RNA Amplification and Labeling System (NuGEN Technologies Inc., San Carlos, CA) according to the manufacturer’s protocol. RNA amplification and hybridization was performed by the University of Michigan DNA Sequencing Core using the Affymetrix Zebrafish Genome Array measuring over 14,900 transcripts (Affymetrix, Santa Clara, CA). Intensities of target hybridization to respective probe features were detected by laser scan of the array. Image files were analyzed using a local version of the GenePattern genomic analysis platform from the Broad Institute (Reich et al., 2006). The samples were Robust Multi-array Average (RMA) normalized and the microarray quality was assessed using the probe-level modeling and quality metrics provided by the Affy package of BioConductor (Irizarry et al., 2003). Intensities from duplicate arrays were averaged (Pearson correlation >0.99 between duplicate arrays). Transcripts with a minimum fold expression change of two were selected as differentially expressed genes. Only the transcripts with the valid Entrez gene ID were used for further analyses. The Database for Annotation, Visualization and Integrated Discovery (DAVID) (http://david.abcc.ncifcrf.gov) (Huang da et al., 2009a; Huang da et al., 2009b) was used to identify over-represented GO terms and KEGG pathways among the differentially expressed genes.

### mRNA qRT-PCR validation

Real-time quantitative RT-PCR was performed using a Bio-Rad iCycler as previously described (Todd et al., 2010). All values were normalized to 18S or GAPDH expression determined in parallel. Standard curves for each primer pair were utilized to confirm linearity among samples. Primers used were as follows: tnnt3a (F, 5′-CAGCTACCTGCAGAAGGCTGACTC-3′; R, 5′-CCTGCGGGGCGATGCACTTAC-3′), mylz2 (F, 5′-CATACC-GTCTCGACATGGCACCC-3′; R, TGCGGTGAACCTGTCGCACT-3′), mylz3 (F, 5′-TCTGCTGACGACATGGCCAACAA-3′; R, 5′-GACGCCAC-CGGTCGGAACAA-3′), cacng1 (F, 5′-TGCACAAGCTGCACTGAG-CCCT-3′; R, 5′-GGCACACAGACCTGCAAAAGCGAA-3′), actc1b (F, 5′-AAACCAACCATGTGTGACGACGAGG-3′; R, 5′-ACAATACCGGTG -GTACGGCCG-3′) and sesn3 (F, 5′-GAGTTCTGCCCGCAACGGCT-3′; R, 5′-ATCAAACCCTGCAGACACCACCA-3′).

### Statistical analysis

Statistical analysis was performed using the Prism GraphPad software package. For analysis of morphologic phenotypes, a Kruskal-Wallis one-way analysis of variance was performed with secondary analysis for differences between specific subsets using a Mann-Whitney *U*-test. For behavioral assays, a one-way ANOVA was performed when multiple groups were analyzed, with post-hoc analysis using a Student’s *t*-test.

## Supplementary Material

Supplementary Material
