# Supplementary material for: Transcriptional changes and developmental abnormalities in a zebrafish model of myotonic dystrophy type 1
Source: Dis Model Mech. 2013 Oct 2;7(1):143–55. doi: 10.1242/dmm.012427 (PMC3882056; doi:10.1242/dmm.012427)
Supplement: Supplementary Material [file supp_7_1_143__index.html]

Transcriptional changes and developmental abnormalities in a zebrafish model of myotonic dystrophy type 1 — Transcriptional changes and developmental abnormalities in a zebrafish model of myotonic dystrophy type 1 — Supplementary Material 

# Transcriptional changes and developmental abnormalities in a zebrafish model of myotonic dystrophy type 1

## DMM012427 Supplementary Material

**Files in this Data Supplement:**

- **Supplementary Material PDF**
