# Supplementary material for: Transcriptional changes and developmental abnormalities in a zebrafish model of myotonic dystrophy type 1
Source: Dis Model Mech. 2013 Oct 2;7(1):143–55. doi: 10.1242/dmm.012427 (PMC3882056; doi:10.1242/dmm.012427)
Supplement: Supplementary Material [file supp_012427_DMM012427.pdf]

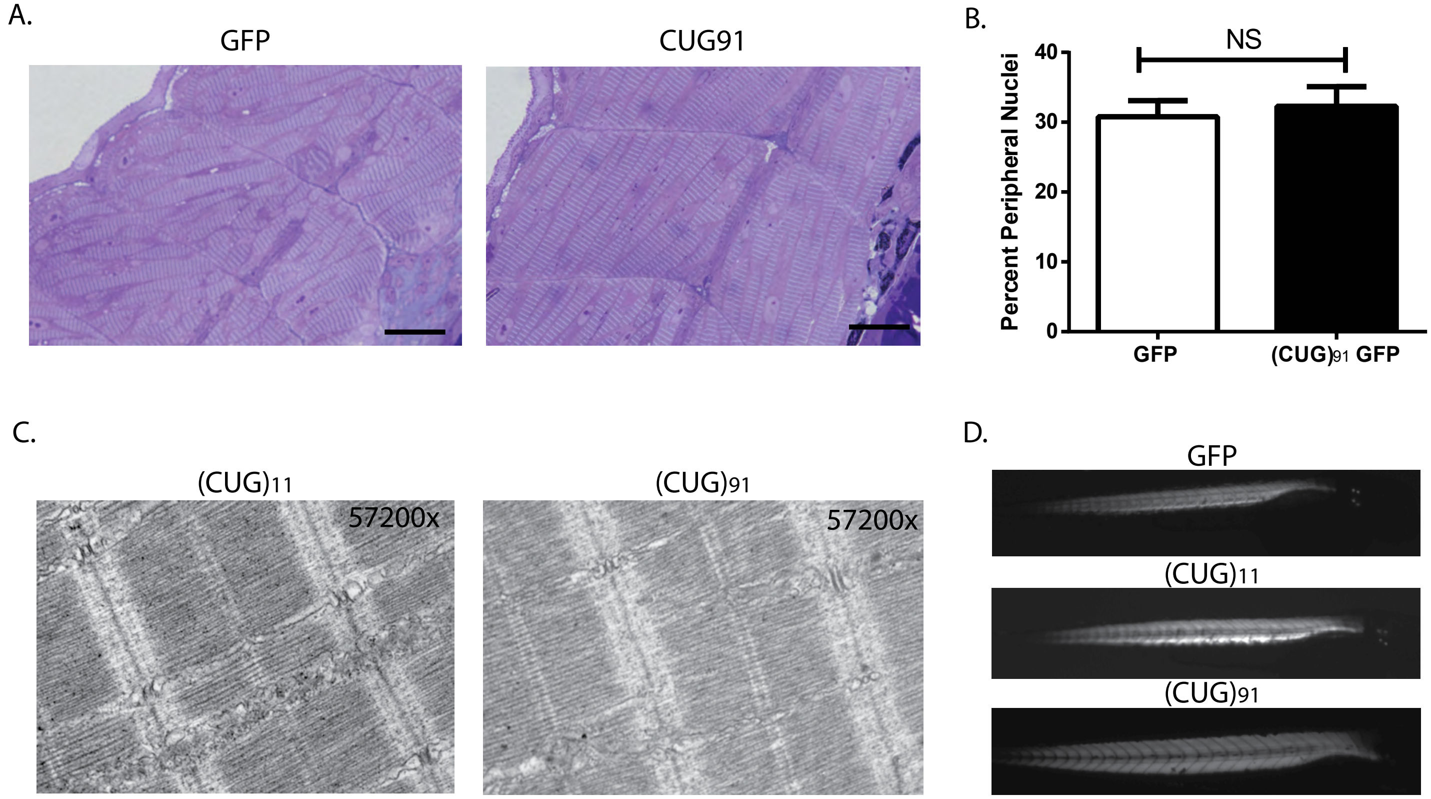

Supplemental Fig. 1. Light and Electron Microscopic Analysis of muscle from GFP$(CUG)_{91}$ mRNA injected embryos. (A) Representative semi-thin longitudinal. muscle sections from 48hpf embryos injected with GFP or GFP$(CUG)_{91}$ mRNA. The scale bar is 20uM. (B) Quantification of nuclear position within muscle fibers in GFP and GFP$(CUG)_{91}$ mRNA injected embryos at 48hpf. NS is not significant. (C) Transmission Electron Microscopy images of GFP $(CUG)_{11}$ or GFP$(CUG)_{91}$ mRNA injected embryos at 48hpf demonstrating normal cyto-architecture. (D) Birefringence images from GFP mRNA, GFP$(CUG)_{11}$ mRNA or GFP$(CUG)_{91}$ mRNA injected embryos at 48h.

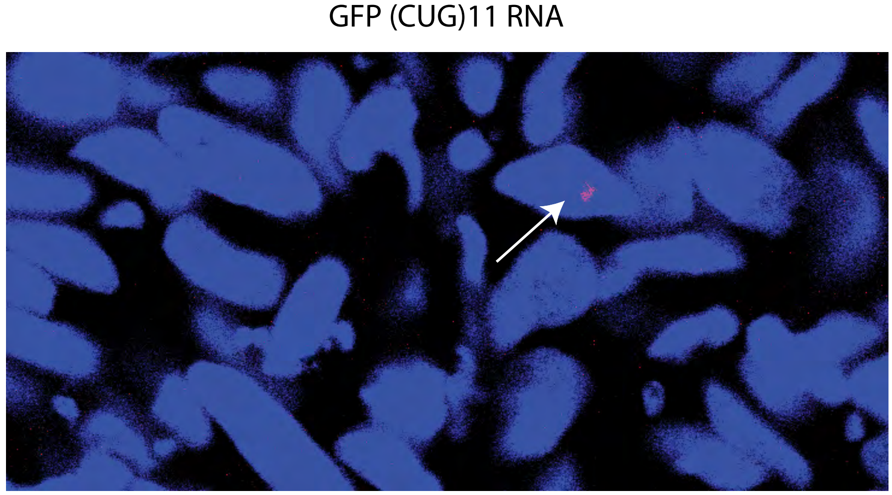

Supplemental Fig. 2. RNA foci in GFP$(CUG)_{11}$ mRNA injected embryos. Representative image of a rare RNA foci (arrow) observed in dissociated myofibers from GFP $(CUG)_{11}$ mRNA injected embryos at 24hpf.

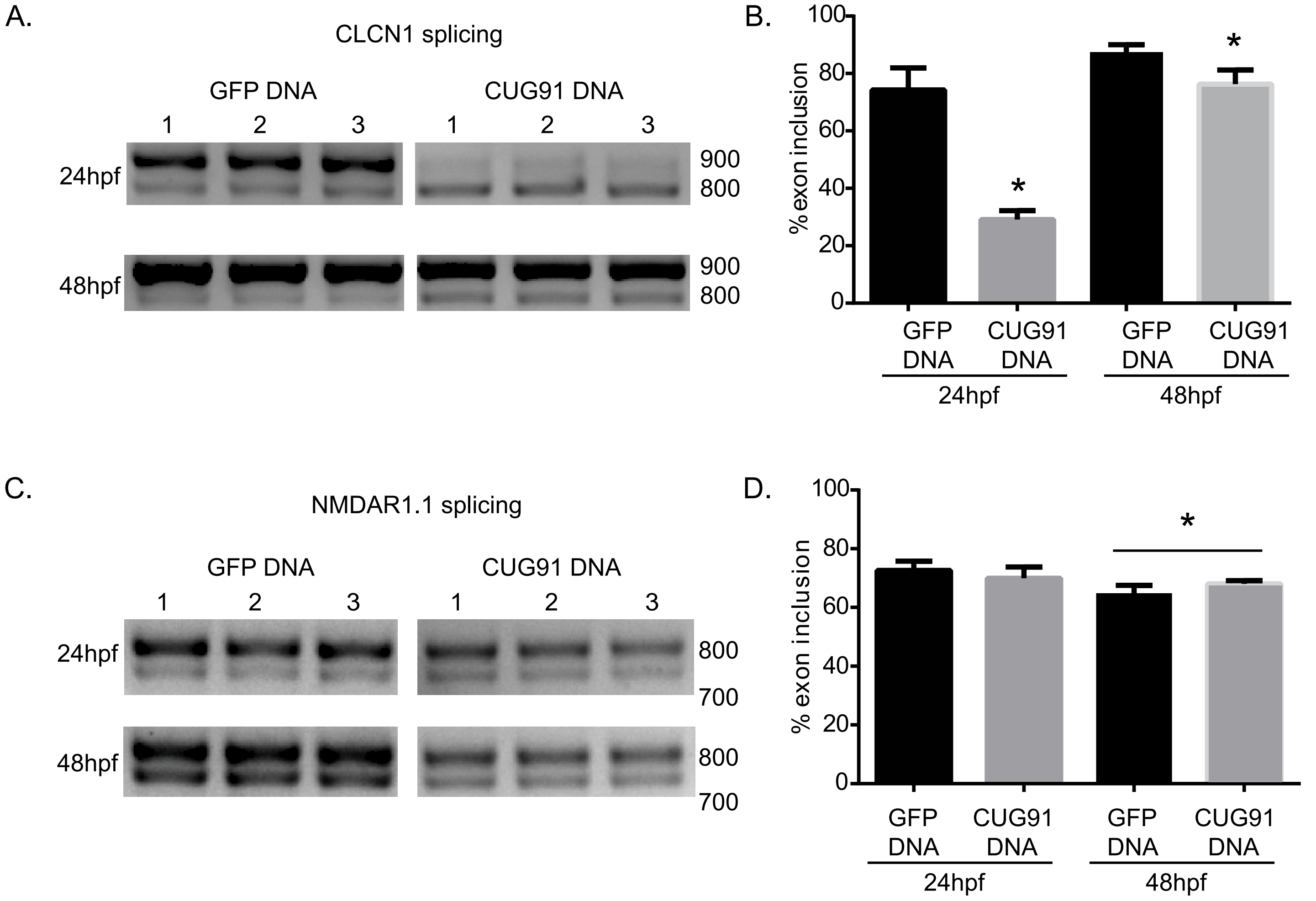

Supplemental Fig. 3. Altered splicing in GFP(CUG)$_{91}$ DNA injected embryos. (A) Representative image of clcn1 splicing at 24hpf (top row) or 48hrs (bottom row) in embryos injected with GFP DNA (left) or GFP(CUG)$_{91}$ DNA (right). Each lane represents a pooled set of 10 embryos from whom RNA was extracted. (B) Quantification of clcn1 splicing in GFP DNA (black bars) or GFP(CUG)$_{91}$ DNA (gray bars) injected embryos at the indicated timepoints. The *y*-axis is the % inclusion of exons 3 and 4 based on densitometry analysis. (C) Representative image of nmdar1.1 splicing at 24hpf (top row) or 48hrs (bottom row) in embryos injected with GFP DNA (left) or GFP(CUG)$_{91}$ DNA (right). (D) Quantification of clcn1 splicing in GFP DNA (black bars) or GFP(CUG)$_{91}$ DNA (gray bars) injected embryos at the indicated timepoints. The *y*-axis is the % inclusion of exon 5 based on densitometry analysis. For B and D, N=5/group, *represents P<0.05 on students t-test.

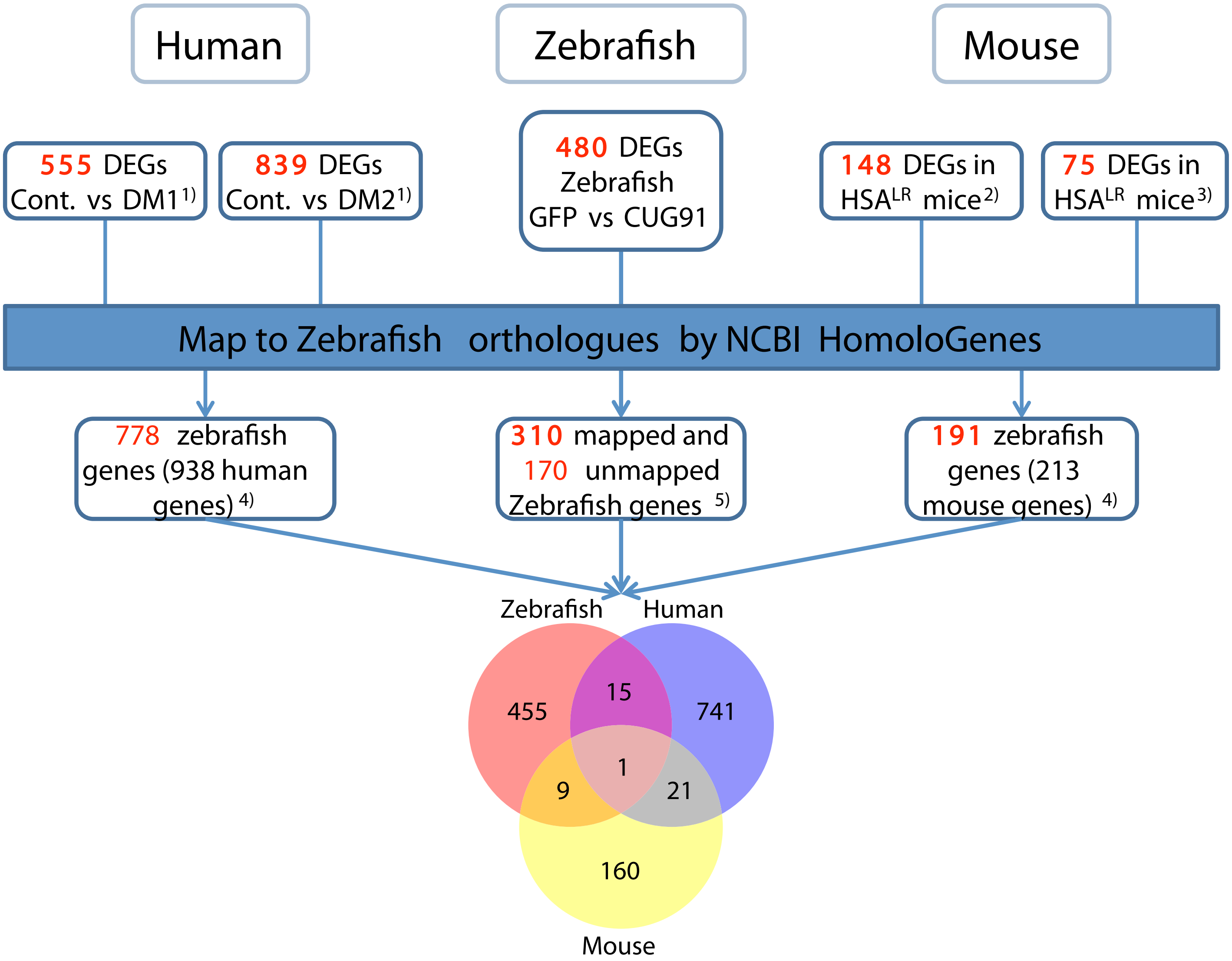

Supplemental Fig. 4. Comparison of differentially expressed transcripts in DM1 models across species. To determine if the transcriptional changes we observed in association with GFP(CUG)$_{91}$ mRNA expression were shared with previous studies in mammals and patient samples,, we reanalyzed published data from 3 studies and determined the zebrafish orthologous for each altered gene. For studies in HAS^{LR} mice(40,41) differentially expressed genes (DEGs) were obtained from the paper, reflecting fold changes of 1.5-2. For the human study of DM1 and DM2 muscle (49), the raw microarray data were downloaded from the NCBI GEO database and processed using our in-house pipeline with a DEG criteria of FDR <0.05 and FC >2. Only human and mouse genes that mapped to the annotated Zebrafish transcriptome were included in the analysis. The Venn diagram shows the degree of overlap of among species for the different models.

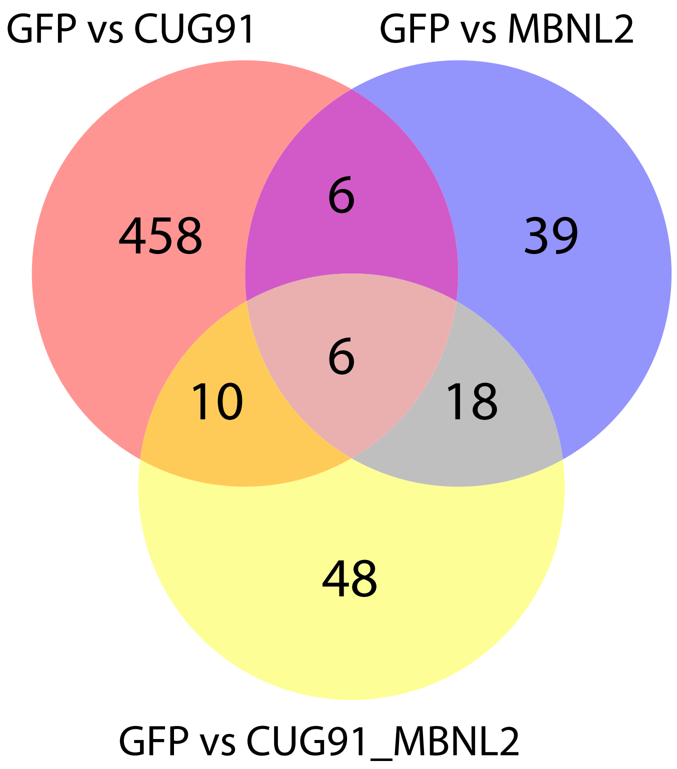

Supplemental Fig. 5. Venn diagram of differentially expressed genes in different fish genotypes. Expression of MBNL2 mRNA in isolation led to a limited number of DEGs when compared to GFP alone. To determine if these genes might be differentially regulated by MBNL2 compared to GFP$(CUG)_{91}$ RNA, we generated a Venn Diagram of differentially regulated genes for each of 3 genotypes compared to GFP. There were no transcripts that showed clear dissociated regulation of transcript expression in the setting of MBNL2 alone compared to GFP$(CUG)_{91}$ mRNA.

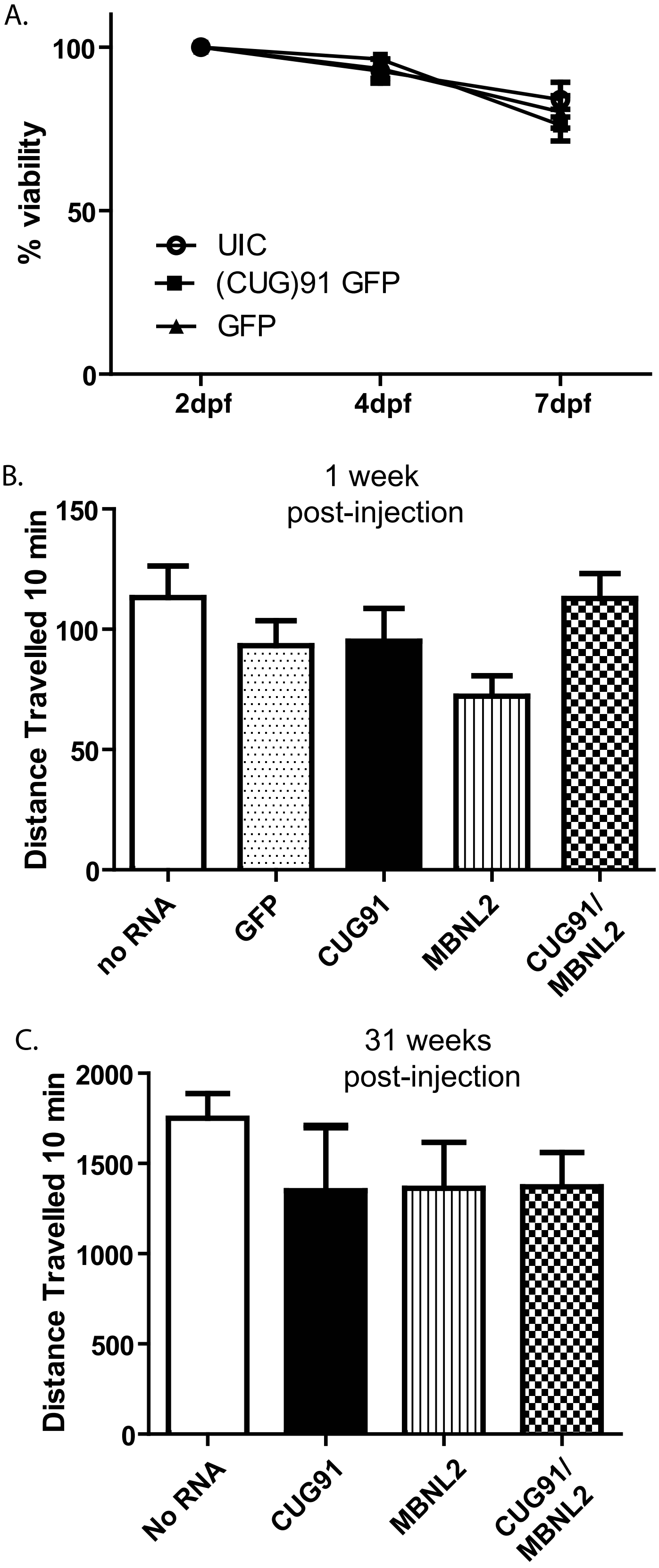

Supplemental Fig. 6. GFP(CUG)$_{91}$ mRNA injected embryos have normal motor phenotypes as adults. (A) Embryos injected with the indicated RNAs that survived to 48hpf were followed for 1 week post fertilization to determine their viability. Error bars represent the 95% Confidence interval. (B) Activity monitoring of zebrafish embryos one week after injection with the indicated RNAs. (C) Activity monitoring of adult zebrafish 31 weeks after injection with the indicated RNAs. For B and C, the graph expresses the total distance travelled in cm in a 10 minute interval. Error bars are SEM. No differences between groups were significant after corrections were made for multiple comparisons.

Group1	Group2	Transcripts (FC > 2.0)	Transcripts without Entrez ID	Transcripts with Entrez ID	Unique Entrez Genes
**GFP**	**CUG91**	747	91	656	480
**GFP**	**MBNL2**	103	13	90	69
**GFP**	**CUG91_MBNL2**	142	21	121	82
**CUG91**	**CUG91_MBNL2**	816	102	714	561

Supplemental Table 1. Differences in zebrafish cDNA microarray across embryos injected with different RNAs. Comparison of cDNA microarray profiles between 24hpf embryos injected with different *in vitro* transcribed RNAs (GFP as a negative control, GFP$(CUG)_{91}$, MBNL2, or GFP$(CUG)_{91}$ and MBNL2 together). The numbers of transcripts which show at least a 2 fold difference in expression between are shown for each comparison.

Supplemental Table 2. cDNA microarray data for embryos injected with different RNAs.

Download Table S2
